# A Tissue Dielectric Constant Evaluation of Knee Edema: A Retrospective Intra-rater Reliability and Association Study of Repeated Measures

**DOI:** 10.7759/cureus.42089

**Published:** 2023-07-18

**Authors:** James P Suchy, Ward M Glasoe, Linda A Koehler

**Affiliations:** 1 Department of Rehabilitation Medicine, Division of Physical Therapy, University of Minnesota, Minneapolis, USA; 2 Masonic Cancer Center, University of Minnesota, Minneapolis, USA

**Keywords:** evaluation, circumferential girth measurement, knee edema, tissue dielectric constant (tdc), orthopedics

## Abstract

Background: This research compared the reliability and association of tissue dielectric constant (TDC) measures of knee edema to circumferential measurements of knee girth recorded as part of a physical therapy examination.

Methods: Twenty adults having observable unilateral knee edema were enrolled. A single examiner measured edematous knee swelling with a TDC device and a tape measure across two visits. The presence of edema was recorded as a positive number in reporting side-to-side differences and a positive percentage in documenting change over time. Intra-rater reliability of the measures was assessed with an intra-class correlation coefficient (ICC). Percent change in edema was evaluated independently for both methods using a paired *t*-test, and the association between measures was assessed by a Pearson’s statistic.

Results: Both measures were reliable (ICC ≥ 0.81), and both detected a significant percentage decrease (*p* < 0.05) in edema across visits. The TDC measure changed by 8.3%, an amount nearly four times larger compared to knee girth (2.4%). The subsequent follow-up comparison revealed an inverse relationship (*p* = 0.049; *r* = -0.44) between the two percent change measurements of edema.

Conclusion: The two methods capture different physical attributes of edema. The TDC records the water content of the tissue, while the use of a tape measure records circumferential limb girth. The TDC measurement was reliable and more responsive in detecting a percentage decrease in knee edema in comparison to a circumferential measure of knee girth. The TDC method may have wider use in directly measuring edema in other tissue structures and regions of the body.

## Introduction

Excess fluid that collects in the body tissues is called edema. Although inflammation that develops following acute trauma facilitates tissue repair and healing, a prolonged period of tissue fluid retention impedes healing and increases the risk of infection [1]. For this reason, chronic edema, defined as edema that lasts longer than three months following injury or secondary to disease processes [2], is measured by various means to document the severity of the condition and monitor the progression of care [3-6].

Edema retained in a limb can be indirectly measured as limb girths with a flexible tape measure [6-9]. The measure, acquired at selected sites along the limb, is reliable and quick to perform, and if one limb is affected, a side-to-side comparison allows for the collection of fluid to be quantified [8]. However, this measurement is limited when both limbs are affected, or in the assessment of certain body parts such as the trunk, neck, or face, or in detecting edema that collects in specific tissues such as a tendon sheath or a bursa (e.g., tendinitis, bursitis).

Research that studies lymphedema (edema associated with inadequate lymphatic drainage) monitors peripheral edema with a tissue dielectric constant (TDC) device [3,4,10-12]. The device measures local tissue water content by emitting a 300 MHz electromagnetic wave to the tissues through a transducer held in contact with the skin over an area of interest [12]. The emitted electromagnetic wave returns to the transducer head, and a reflection coefficient is calculated determining the TDC, with the TDC value displayed on the unit’s screen [12]. Recorded TDC values range from 0 to 80 and are reflective of the amount of water present under the localized area. A TDC value of zero is indicative of no water, and a value of 78 is reflective of pure water [13]. The value is a unitless number corresponding to the ratio of tissue permittivity to vacuum permittivity [13].

The MoistureMeterD device, manufactured by Delfin Technologies (Kuopio, Finland), measures the water content of body tissues [4]. This portable TDC device has an effective penetration depth of approximately 2.5 mm, which is a relevant depth of edematous superficial tissue [13]. Since a TDC device directly measures the water content of tissues, research is needed to evaluate its use in determining edema that develops with injury or disease states, because orthopedic conditions account for half of all healthcare encounters in the United States [14].

The aim of this study was to measure knee edema and examine the reliability, responsiveness, and association of the TDC in comparison to knee girth measures recorded as part of routine physical therapy examination. The two measures were hypothesized to have a positive association. Ultimately, this investigation was premised on the idea that if the TDC measurement of knee edema could be demonstrated reliable, the TDC device may detect edema in any anatomic structure or body region.

## Materials and methods

Study design

This was an observational retrospective cross-sectional study that followed the Guidelines for Reporting Reliability and Agreement/Association Studies (GRRAS) [15].

Participants

Included were adults (18 and older) having unilateral knee injury or surgery who were treated in physical therapy with the presence of visible edema in the affected limb. The knee was evaluated because edema of the knee is easy to observe and palpate following injury or surgery [9], because the measure of knee girth is highly reliable [5,6,9], and because a 1.0 cm side-to-side difference in the circumferential knee girth is considered clinically meaningful in documenting care [8,9]. Edema was measured as circumferential knee girth and with the TDC at initial examination and again at follow-up and having a side-to-side difference in knee girth (at initial examination) of 1.0 cm or larger. Excluded were obese individuals (body mass index ≥ 30), as well as those with a history of previous knee injury or systemic disease, which could potentially mask or confound the measurement. All enrollment decisions and measurements were made by a single physical therapist (JS) who had 19 years of experience treating an orthopedic caseload. The timing of the follow-up visit was not controlled but was instead determined at the discretion of the clinician. The demographic attributes of the sample accessed from the chart review included age, sex, diagnosis of surgery or acute injury, side of injury, and the count of days between visits.

The data of 20 patients (of 29 consecutive cases) seen in physical therapy following a knee injury or surgery were studied from a medical record review. An a priori power analysis indicated a t-test paired sample of 20 participants would have 80% power to detect a difference of 5 (unitless) in TDC measures of knee edema across two visits. The nine cases of the medical charts reviewed but not studied were because edema was measured only at the initial examination (N = 6), or the side-to-side difference in knee girth recorded at the initial examination (N = 3) did not reach the 1.0 cm threshold required for enrollment. All charts reviewed had a statement signed by the patient granting approval for the use of their data in research. This study also underwent review by a University Institutional Review Board (ID: STUDY00008075) and received an exemption determination.

Procedures

**Figure 1:**
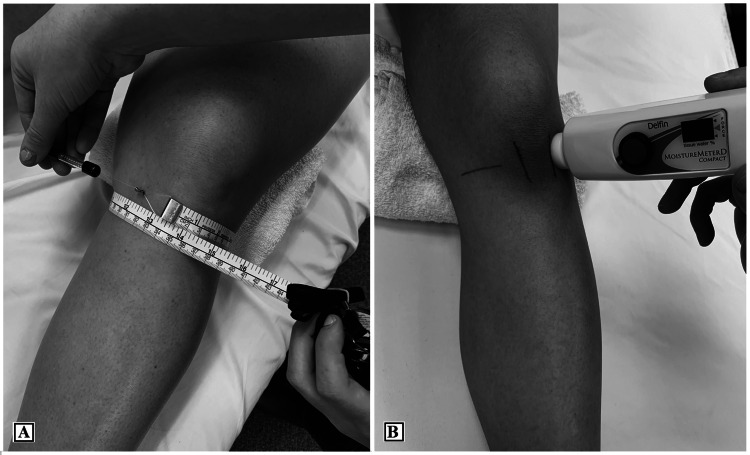
The measurement methods: (A) Tape measurement of the knee girth. (B) TDC measure of the knee local tissue water content

Knee girth and TDC measures were made bilaterally at the initial examination and again at follow-up with the patient positioned supine and consistently using the same-sized towel roll placed under their knee (Figure 1). Edema was reported as a positive number in recording side-to-side differences and as a positive percentage in documenting a change across visits (i.e., percent change in edema = X involved - X uninvolved / X uninvolved x 100). The percent change calculation in essence normalized the data allowing for the two measurements to be compared. Therefore, if there was a reduction in girth or TDC measures, the decrease would be documented as a positive percent reduction.

Knee girth was measured using a Gullick tape (FitnessMartW, Gays Mills, WI, USA). This tension-gauge tape measure conforms to the body and features a spring that provides consistent loading tension [5,7]. The tape measure (Figure 1A) was placed around the joint line, and the value was recorded to the nearest 0.1 cm. A tension gauge tape measure has been reported to be more reliable than a standard tape measure in recording limb girth [7].

The TDC device measures were acquired medial and lateral to the patella tendon (Figure 1B), with the average recorded as a single value at each knee. The TDC measurement was made by holding the device perpendicular to the limb, its probe pressed onto the skin with consistent pressure for five seconds, and the value recorded displayed on the device. Prior to using the TDC device to measure knee edema in practice, the single examiner received instruction on its use from the senior author (LK) who has extensive experience in measuring lymphedema in research [10,11]. TDC measurements of the lower leg have been demonstrated reliable [3] but no previous study has reported measures at the knee.

Data analysis

Descriptive statistics analyzed the demographic attributes of the sample. Intra-class correlation coefficient (ICC) and standard errors of measurement (SEMs) were computed on data collected from the uninvolved knee across visits (initial exam and follow-up) to evaluate the reliability of the knee girth and TDC measurements. An ICC value approaching 1.00 indicated near-perfect agreement between trials. The SEM estimated the extent of expected error associated with obtaining the measure, expressed in the unit of measurement (cm), and indicates the range of values expected when the measure is repeated. The side-to-side difference in measures was tracked descriptively across visits and as a percent change in edema, with this variable evaluated independently for both methods using a paired t-test and for the association by a Pearson’s statistic. Significance was set at p < 0.05 for all analyses.

## Results

Table 1 reports the demographic attributes of the sample. The patients were young (mean age 24), half were females, 65% had knee surgery and 35% had an undiagnosed knee injury, and the time interval between the initial examination and follow-up averaged 20 days.

**Table 1:** Demographic attributes n: number of participants, SD: standard deviation

Sample	Age, years mean ± SD (range)	Surgery n (%)	Undiagnosed knee injury n (%)	Right side n (%)	Time to follow-up, days mean ± SD (range)
n = 20 (10 males)	24 ± 8.3 (18 to 53)	14 (65%)	6 (35%)	9 (45%)	20 ± 12.8 (2 to 63)

Table 2 reports the knee edema measurements recorded at the initial examination and at follow-up. Reliability of knee girth, repeatedly measured on the uninvolved side, resulted in an ICC of 0.98 with a SEM of 0.66 cm; TDC measurements had an ICC of 0.81 with a SEM of 6.74. Regardless of methods, the measure of edema (Table 2) decreased over the course of physical therapy visits.

**Table 2:** Knee edema measurement values displayed by method TDC: tissue dielectric constant, SEM: standard error of the mean

	Knee girth, cm mean ± SEM (range)		TDC measure, unitless mean ± SEM (range)	
	Swollen knee	Non-injured side	Swollen knee	Non-injured side
Initial exam	37 ± 0.87 (32 to 48)	35 ± 0.78 (30 to 43)	45 ± 2.07 (23 to 64)	32 ± 2.53 (7 to 47)
Follow-up	36 ± 0.87 (30 to 46)	35 ± 0.73 (30 to 42)	41 ± 2.53 (12 to 66)	32 ± 2.46 (9 to 48)

**Figure 2:**
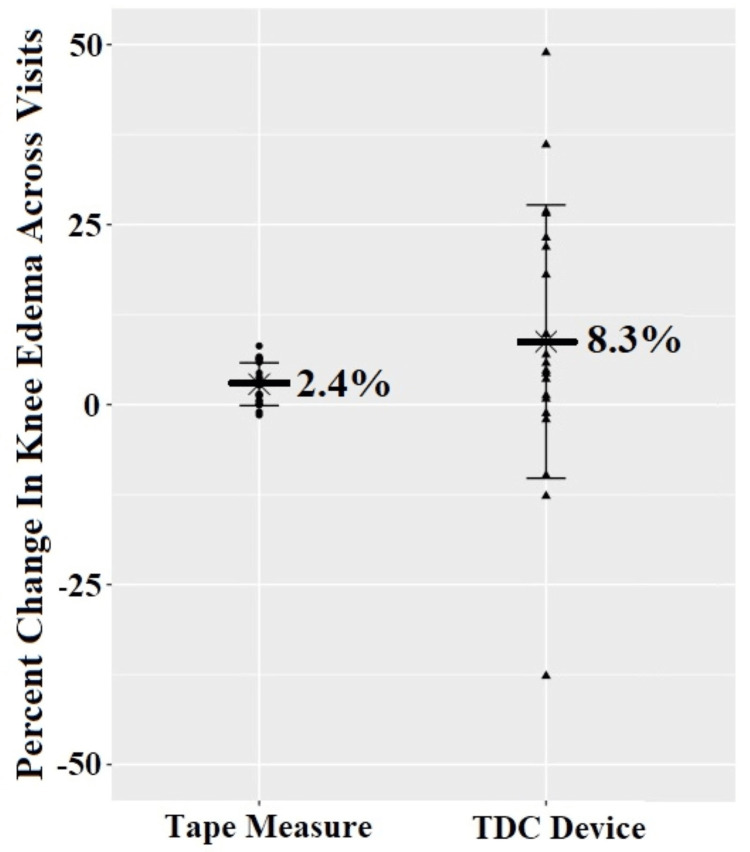
Percent change in knee edema on patients (n = 20) recorded with the tape measure compared to a TDC measuring device. Data is displayed relative to a 0 (no change) baseline. A positive value indicates a decrease in knee edema across visits. The mean value of the measurement is displayed with standard deviation represented by error bars TDC: tissue dielectric constant

The side-to-side difference in the Gulick knee girth tape measures averaged 2.3 cm at the initial examination, decreasing to 1.3 cm at follow-up, whereas the side-to-side difference in TDC measurement averaged 13.2 at the initial exam, decreasing to 8.8 at follow-up (Table 3). The decrease, calculated as a positive percent reduction, was significant in knee girth (p = 0.001) and for the TDC (p = 0.049), with knee girth detecting a smaller change (2.4% reduction) compared to the TDC (8.3% reduction) across visits (Figure 2). The subsequent follow-up comparison revealed an inverse relationship (p = 0.049; r = -0.44) between the two percent change measurements. While decreased, the unilateral presence of knee edema did not fully resolve during the enrollment period (Table 3).

**Table 3:** Side-to-side difference in edema measurements displayed by method. A positive value indicates the presence of edema in the involved knee TDC: tissue dielectric constant, SEM: standard error of the mean

	Knee girth, cm mean ± SEM (range)	TDC measure, unitless mean ± SEM (range)
Initial exam	2.3 ± 0.25 (1.0 to 4.9)	13.2 ± 2.45 (2.0 to 41.5)
Follow-up	1.3 ± 0.29 (-1.0 to 3.8)	8.8 ± 2.08 (-1.0 to 29.5)

## Discussion

The TDC device was originally developed to quantify lymphedema, but recently, research has begun to study its effectiveness in quantifying other forms of interstitial edema [3,4]. To this end, this study acquired TDC measures of knee edema and explored the association between the TDC measurement and circumferential knee girth. In evaluating this comparison, one should weigh with caution the validity of quantifying knee girth to represent the retention of fluid, as the measurement only indirectly captures the construct. Nevertheless, in accepting that limb size increases when edema is retained and decreases when edema is reduced, the measurement of girth, on its face value, is a useful surrogate for objectifying the presence of knee edema [5,6,9]. Furthermore, studies have validated a decrease in TDC values to represent a reduction in local measurement of changes in tissue water in human skin [13].

Comparison of measurements

The patients studied represent a cross-section of adults (age 18 to 53) treated in physical therapy following a knee injury or surgery. The mean girth (Table 2) measured on the involved (37 cm) and uninvolved (35 cm) knees at the initial exam are within 1.5 cm of the mean values reported in a previous study [6] of adults treated following anterior cruciate ligament surgery.

The TDC measurement recorded on the uninvolved knee was 32 (Table 2). Although no previous study has reported TDC measures at the knee, reports indicate that expected TDC values depend on the body part measured [16,17] and the type of edema (i.e., acute vs. chronic) [11,13,18-21]. At least two studies [3,4] recorded TDC measures distal to the knee at an effective depth of 2.5 mm. One study [3] measured 34 healthy women, reporting mean values of 38 at the foot, 29 at the ankle, and 31 at the calf. Another study [4] acquired the measure on the foot and along the medial side of the lower leg in 88 healthy adults (44 women), reporting a collective mean of 31. These results suggest the mean of 32 recorded in this study on the uninvolved knee falls within the expected range. Adding further credibility to the data we report, the side-to-side difference recorded with both methods decreased across visits (Table 3) to correspond with the patient’s recovery. While both measurements decreased, the presence of knee edema did not resolve fully during the enrollment period (Tables 2 and 3) which may be due to the variability in the healing process and the type of edema.

Reliability was assessed by repeating the measurements on the uninvolved knee at the initial examination and again at follow-up. The measure of knee girth was highly reliable, with an ICC = 0.98 and a SEM = 0.66 cm. An ICC gives a comparison of two or more repeat measures. A SEM of 0.66 cm indicates that the error of the measure was smaller in magnitude than the 1.0 cm side-to-side difference deemed clinically meaningful in identifying the presence of knee edema [8,9].

The TDC measurements were also reliable, with an ICC = 0.81 and a SEM of 6.74. A SEM of 6.74 is about 10% of the total range (from 9 to 66) of the TDC values recorded in the patients sampled (Table 2). To illustrate the impact of this measurement error, an examiner who recorded a mean value of 32 and a SEM of 6.74 has a 68% chance the true score lies within ±1 SEM or a TDC range from 25.26 to 39.74. Consequently, the TDC mean of 45 measured on the involved knee at the initial exam (Table 2) represents a meaningful side-to-side difference because the value of 45 falls outside the range of expected values recorded in this study.

The knee girth and TDC measures had a significant negative relationship (p = 0.049; r = -0.44). This result is surprising, especially when considering that both measures decreased across visits (Tables 2, 3). One explanation could be the variability of the TDC measurements (Figure 2). The spread in the distribution of data indicates it is possible that a large decrease in edema recorded with the TDC device could correspond with an increase in knee girth recorded with a tape measure in some of the patients sampled or vice versa. Knee girth accounts for all of the tissue (i.e., edema, muscle, bone, and adipose). TDC accounts for both free and bound water in a localized area located 2.5 mm underneath the transducer head touching the skin. Therefore, the change in muscle size or in the patient’s weight may have been a confounding variable. Recall, the time to follow-up was not controlled in this study but was instead left to the discretion of the examiner. Since follow-up occurred at an average of 20 days after the initial examination (Table 1), muscles that surround the knee may have hypertrophied as the patient recovered. While hypertrophy of the muscles would increase girth, it would not influence the TDC measurement. An increase in body weight, posterior knee edema, and hypertrophy of the muscle and tendon (i.e., hamstring and gastrocnemius) may also explain why the measurement of knee girth detected a smaller percent decrease change (2.4%) across the visits (Figure 2) as compared to the 8.3% detected by the TDC measurement over the same time period. In addition, our study measured localized edema medial and lateral to the patellar tendon only and, therefore, would not have captured posterior knee edema if present. Since the girth measures account for all tissue, the girth measures may have also been measuring posterior knee edema if present. Ultimately, the two methods capture different physical attributes of edema, but only the measurement of knee girth is affected by the change in limb size that may be unrelated to the retention of fluid.

Implications for practice

Investigating the TDC measurement of trauma-related knee edema can serve as a starting point for researchers interested in studying acute tissue inflammation and chronic edema that develops following orthopedic trauma and disease. Implications for practice include the assessment of edema that collects in superficial tissues and body structures that are difficult to evaluate with current clinical methods. Edema from specific injuries such as an inflamed tendon or bursa sac can be challenging to detect because the edema is localized. Joint injuries of the jaw, spine, or pelvis are examples of locations that are difficult to measure because circumferential measurements are not useful in these regions. Given these considerations, the TDC has the potential to overcome barriers of current clinical methods, because it has the ability to measure localized edema and measure areas of the body that cannot be quantified with circumferential methods. Therefore, the TDC detection of edema could steer practitioners toward delaying treatment in consideration of referring a patient for imaging or in prescribing medication, steroid injections, and other anti-inflammatory modalities. In cases where TDC measures are not elevated to correspond with a reduction in pain and improved function, strengthening exercises could be started earlier and advanced quicker in the rehabilitation protocol.

Strengths and limitations

Employing a single experienced examiner allowed us to repeatedly deliver the data collection techniques and optimize the selection of patients. Leaving the time-point for acquiring the repeated measures to the discretion of the examiner (i.e., not standardizing the repeated measure time-point) can be viewed as a strength of the study. This meant the initial measurements were taken when visible edema was observed and repeat measures were taken primarily based on convenience (not predetermined intervals but when provider and patient determined a follow-up was needed), although not standardizing the time between visits made it a confounding variable. Despite this reality, good consistency was seen in the directional change in edema recorded by both methods across visits (Tables 1, 2).

The study had other limitations. Both surgical and non-surgical patients having a knee injury were included in the small group sampled, which means the exact location of edema, composition of and type of tissue edema (chronic vs. acute), and scarring may have been different between groups. For example, the TDC had a limited depth of penetration and, therefore, may not have been able to detect edema located in deeper tissue such as intracapsular or bursa edema. In addition, it is uncertain if the difference in fluid content including surgical bleeding may have influenced the TDC measurement, although TDC results responded similarly in both groups. Additionally, acquiring a third measurement at final discharge from physical therapy would have aided in understanding whether the edema ever fully resolved during the time course of treatment. In addition, obese individuals were excluded from the study, and there was a lack of diversity in the group; therefore, the results are not generalizable. Although the literature shows TDC values are not confounded by obesity, differences in TDC values may be dependent on race [22-23]. Lastly, the TDC measurement of knee edema performed in this study is novel, which limited the interpretation of results due to the lack of normative data and the homogeneity of the sample.

## Conclusions

This study compared TDC and circumferential girth measurements of knee edema in 20 patients following a knee injury or surgery. Both measures were demonstrated to be reliable (ICC ≥ 0.81), with the TDC identified as being more responsive in detecting a percentage decrease in edema during the enrollment period. The two methods capture different physical attributes of edema. Because only the circumferential measure of knee girth is influenced by the change in limb size unrelated to fluid retention and because the TDC measures of knee edema were variable (total range from 9 to 53), the association between measures was negative (r = -0.44). Although the TDC shows promise in measuring knee edema following orthopedic injury, future research is needed to explain the inverse relationship between the TDC and knee girth measures over time and investigate potential confounding variables, such as changes in muscle size or patient weight.
